# Distinct systemic immune networks define severe *vs*. mild COVID-19 in hematologic and solid cancer patients

**DOI:** 10.3389/fimmu.2022.1052104

**Published:** 2023-01-09

**Authors:** Flávio Pignataro-Oshiro, Amanda B. Figueiredo, Nayane A. L. Galdino, Katia L. P. Morais, Walderez O. Dutra, Bianca Grassi de Miranda Silva, Diego Feriani, Flávia de Azevedo Abrantes, Ivan Leonardo Avelino França e Silva, Jayr Schmidt Filho, Juliana Valéria de Souza Framil, Marcelle Goldner Cesca, Rachel Simões Pimenta Riechelmann, Marjorie V. Batista, Kenneth J. Gollob

**Affiliations:** ^1^ International Research Center, Translational Immuno-oncology Group, A.C.Camargo Cancer Center, São Paulo, Brazil; ^2^ Department of Morphology, Federal University of Minas Gerais, Belo Horizonte, Brazil; ^3^ Infectious Diseases Department, A.C.Camargo Cancer Center, Sao Paulo, Brazil; ^4^ Hematology Department, A.C.Camargo Cancer Center, Sao Paulo, Brazil; ^5^ Clinical Oncology Department, A.C.Camargo Cancer Center, Sao Paulo, Brazil; ^6^ Hospital Israelita Albert Einstein, Israelite Institute for Education and Research, Translational Immuno-oncology Laboratory, São Paulo, Brazil

**Keywords:** SARS-CoV-2, COVID-19, cancer, systemic immune profile, disease severity prediction, cytokine-release syndrome

## Abstract

**Introduction:**

The COVID-19 pandemic, caused by the coronavirus SARS-CoV-2, has impacted health across all sectors of society. A cytokine-release syndrome, combined with an inefficient response of innate immune cells to directly combat the virus, characterizes the severe form of COVID-19. While immune factors involved in the development of severe COVID-19 in the general population are becoming clearer, identification of the immune mechanisms behind severe disease in oncologic patients remains uncertain.

**Methods:**

Here we evaluated the systemic immune response through the analysis of soluble blood immune factors and anti-SARS-CoV-2 antibodies within the early days of a positive SARS-CoV-2 diagnostic in oncologic patients.

**Results:**

Individuals with hematologic malignancies that went on to die from COVID-19 displayed at diagnosis severe leukopenia, low antibody production against SARS-CoV-2 proteins, and elevated production of innate immune cell recruitment and activation factors. These patients also displayed correlation networks in which IL-2, IL-13, TNF-alpha, IFN-gamma, and FGF2 were the focal points. Hematologic cancer patients that showed highly networked and coordinated anti-SARS-CoV-2 antibody production, with central importance of IL-4, IL-5, IL-12A, IL-15, and IL-17A, presented only mild COVID-19. Conversely, solid tumor patients that had elevated levels of inflammatory cytokines IL-6, CXCL8, and lost the coordinate production of anti-virus antibodies developed severe COVID-19 and died. Patients that displayed positive correlation networks between anti-virus antibodies, and a regulatory axis involving IL-10 and inflammatory cytokines recovered from the disease. We also provided evidence that CXCL8 is a strong predictor of death for oncologic patients and could be an indicator of poor prognosis within days of the positive diagnostic of SARS-CoV-2 infection.

**Conclusion:**

Our findings defined distinct systemic immune profiles associated with COVID-19 clinical outcome of patients with cancer and COVID-19. These systemic immune networks shed light on potential immune mechanisms involved in disease outcome, as well as identify potential clinically useful biomarkers.

## Introduction

Coronavirus Disease 2019 (COVID-19) is an illness caused by Severe Acute Respiratory Syndrome Coronavirus-2 (SARS-CoV-2), identified following an outbreak of unknown pneumonia cases during 2019. The etiological agent was classified as a betacoronavirus closely related to Severe Respiratory Syndrome Coronavirus (SARS-CoV) and Middle East Respiratory Syndrome Coronavirus (MERS-CoV), associated with outbreaks in 2002 and 2012, respectively (1).

Patients infected by SARS-CoV-2 primarily present with fever, cough, shortness of breath, dyspnea, lung infiltration and possibly respiratory failure, depending on the disease severity (1). The symptoms are partially explained by the virus’ biology, which leads to infection of cells *via* proteases and angiotensin-converting enzyme 2 (ACE2), present in respiratory tract tissues (1). The acute respiratory distress syndrome observed in COVID-19 is believed to be due to a cytokine-release syndrome, lack of immune regulation, and may be associated with multiple-organ failure (2).

Typically about 80% of the cases are classified as mild, 15% as severe and 5% as critical (1). Nevertheless, data from May 2020 reported that about 90% of the registered cases in Brazil resulted in hospitalization, of which 30% were admitted to intensive care units (ICU) (3). Later on, in 2021, 99% of the cases reported in the Brazilian Healthcare System database (DATASUS) had resulted in hospitalization, of which 36% needed ICU admissions, culminating in a rate of death approximately 37% (4). By October 2022, Brazil had more than 34 million confirmed cases, and almost 688,000 deaths, reaching a lethality of 2% (5). It is important to take into consideration that that Brazil is a country with a unique healthcare system, an expressive prevalence of comorbidities among its population, and that its national vaccination campaign had a very good coverage once implemented, explaining in parts both the high rates of hospitalization and ICU admissions in 2020 and 2021, and the progressively lower infection and lethality rates by 2022 (4, 6).

In general, male individuals aged 60 or above, with chronic cardiovascular or respiratory conditions, hypertension, obesity or diabetes have increased risk of severe disease and death (1, 2). Individuals with cancer specifically present lower survival rates (7, 8) with case-fatality percentages ranging from 11% to 26% (9), and higher chances of hospitalization and death compared to those from the general population (10). In patients with cancer the risk of developing severe COVID-19 is associated with hematologic malignancies, non-white race, age 65 or above, chronic lymphopenia, use of corticosteroid combined with immune checkpoint inhibitors therapy, former or current smoker status, hypertension, and chronic kidney, cardiac or lung disorders (9, 10). Within the hematological malignancies, acute myeloid leukemia, aggressive non-Hodgkin lymphoma, multiple myeloma and plasma cell malignancies were associated with a greater risk of hospitalization and death (10). Similarly, some solid tumor patients also showed higher risk of severe COVID-19 than the general population (8, 11, 12). In Brazil, a study demonstrated that an increased chance of death in cancer patients was associated with lung and hematological tumors (13).

Oncologic patients, and in particular those with hematologic malignancies, often display immunosuppression as a consequence of cancer progression or of therapeutic interventions (14). We hypothesize that specific systemic immune response profiles in cancer patients may be related to distinct COVID-19 clinical outcomes and that these profiles could potentially be used to predict COVID-19 progression. Thus, we investigated systemic soluble factor immunoregulatory networks in oncologic COVID-19 patients with differential clinical outcomes. We identified specific immune mediators and networks associated with hematologic malignancy patients that maintained mild COVID-19 *vs*. those that develop severe disease and death. These findings advance our understanding of immune imbalances related to severe COVID-19 in oncologic patients and point towards potentially clinically useful immune markers of severity in oncological patients.

## Results

### Hematologic or solid tumor cancer patients display distinct outcomes of COVID-19

**Figure 1:**
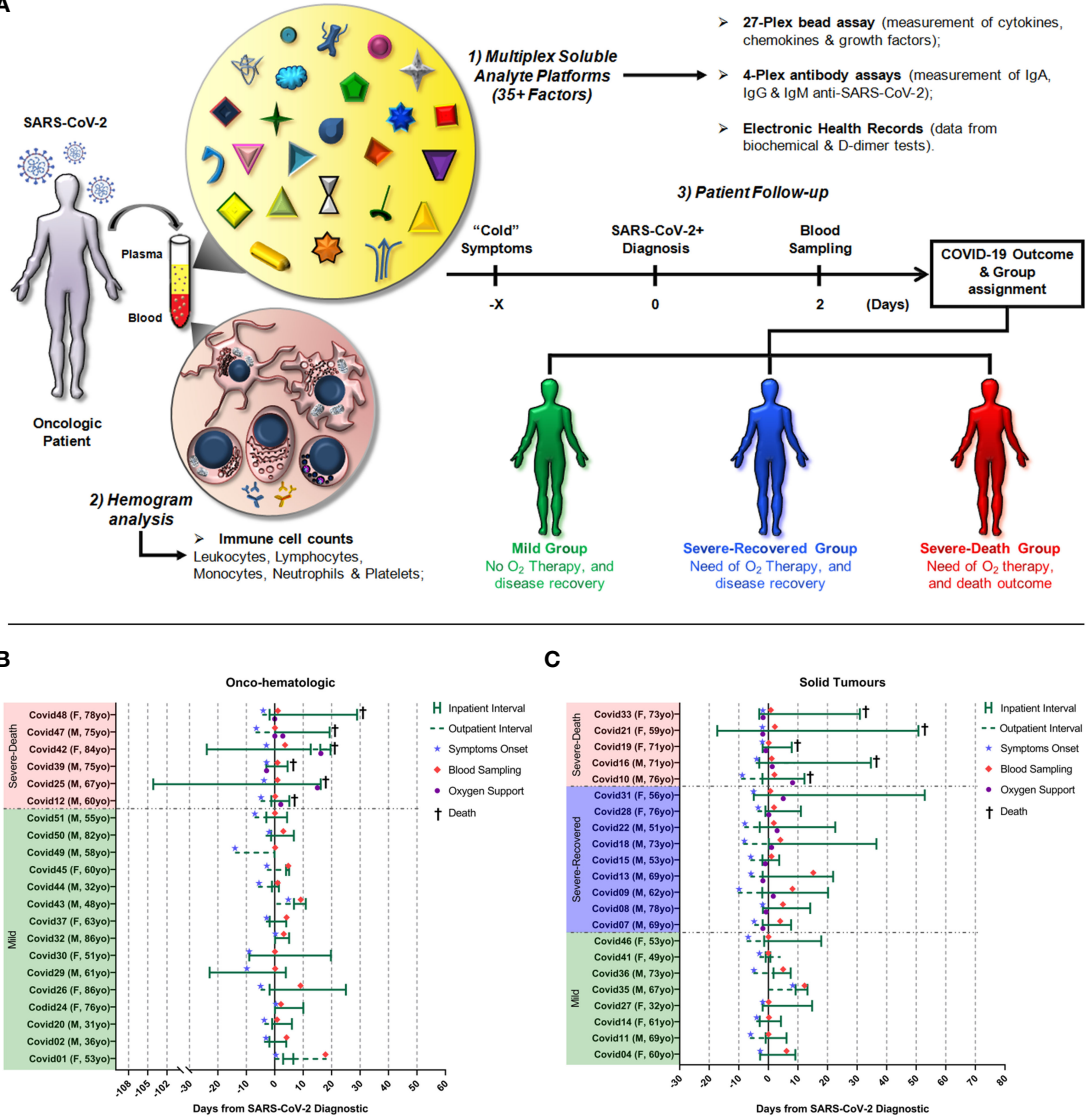
Experimental design, cohort clinical characteristics and timeframe of sampling utilized in the study. The first illustration **(A)** represents the experimental design of this study. Briefly, oncologic patients from A.C.Camargo Cancer Center that had a positive diagnostic of SARS-CoV-2 infection were invited to participate in the study. Blood samples were drawn at a median of two days after the confirmed diagnostic and analyzed for the concentrations of soluble factors and cell counts. Then each patient was followed-up and assigned to a group based on the need of supplemental O_2_ therapy and outcome of COVID-19. Below, the illustration represents the timeframe of hospitalization, symptoms onset, blood sampling and clinical complications (need of oxygen supplementation and death) observed in hematologic cancer **(B)** or solid tumor **(C)** patients. Time zero was defined as the day of the positive SARS-CoV-2 qPCR positive diagnostic.

We separated the oncological patient cohorts into hematologic cancers (n = 21) and solid tumors (n = 22) based on previous studies demonstrating distinct clinical outcomes in cancer patients with COVID-19 (8, 11, 12). We further segregated COVID-19 disease severity in Mild, Severe-Recovered, and Severe-Death depending on the use of supplemental oxygen therapy, based on recommendations by the World Health Organization **(** **Figure 1A** **)**. A summary of patient characteristics, including the timeframe of hospitalization, onset of symptoms, blood sampling and clinical complications observed during COVID-19 is shown **(** **Figures 1B, C** **)**. Individuals with hematologic malignancies displayed a distinct COVID-19 evolution as compared to patients with solid tumors, showing either mild disease or severe disease with progression to death, whereas 41% of the patients with solid tumors developed severe COVID-19 but recovered. Further epidemiological information, such as age and sex distribution, tumor type, treatments, Eastern Cooperative Oncologic Group (ECOG) performance status score and cancer status is presented in **Table 1** and **Supplementary Figure 1**.

**Table 1:** Clinical and demographic characteristics of COVID-19 hematologic cancer and solid tumor patients.

	Onco-hematologic (n = 21)		Solid Tumor (n = 22)		
	Mild (n = 15)	Severe-Death (n = 6)	Mild (n = 8)	Severe-Recovered (n = 9)	Severe-Death (n = 5)
Age					
** Median**	58	75	60.5	69	71
** Range**	31-86	60-84	32-73	51-78	59-76
p-value (median)*	p = 0.0990		p = 0.1459		
** p-value (Fisher)^#^**	**p = 0.0456**		p = 0.9999		
Sex					
** Female**	6 (40%)	2 (33%)	5 (63%)	2 (22%)	3 (60%)
** Male**	9 (60%)	4 (67%)	3 (37%)	7 (78%)	2 (40%)
** p-value (Fisher)^#^**	p = 0.9999		p = 0.6241		
ECOG Score					
** Zero**	12 (80%)	3 (50%)	6 (75%)	6 (67%)	2 (40%)
** I-II**	3 (20%)	2 (33%)	2 (25%)	3 (33%)	2 (40%)
** III-IV**	0 (0%)	1 (17%)	0 (0%)	0 (0%)	1 (20%)
** p-value (Fisher)^$^**	p = 0.2857		p = 0.2273		
Hematological Malignancy					
** Lymphoma**	9 (60%)	2 (33%)	NA	NA	NA
** Leukemia**	2 (13%)	1 (17%)	NA	NA	NA
** Myeloma**	2 (13%)	3 (50%)	NA	NA	NA
** Other hematological**	2 (12%)	0 (0%)	NA	NA	NA
Solid Tumor					
** Breast**	NA	NA	3 (38%)	0 (0%)	0 (0%)
** Digestive Organs**	NA	NA	2 (25%)	1 (11%)	3 (60%)
** Prostate**	NA	NA	2 (25%)	1 (22%)	0 (0%)
** Urinary Tract**	NA	NA	0 (0%)	2 (22%)	2 (40%)
** Lungs**	NA	NA	0 (0%)	2 (22%)	0 (0%)
** Reproductive System**	NA	NA	0 (0%)	1 (11%)	0 (0%)
** Other**	NA	NA	1 (12%)	2 (22%)	0 (0%)
Comorbidities					
** Diabetes**	3 (20%)	2 (33%)	2 (25%)	2 (22%)	2 (40%)
** Obesity**	1 (7%)	1 (17%)	2 (25%)	2 (22%)	0 (0%)
** Cardiac Disease**	6 (40%)	6 (100%)	4 (50%)	7 (78%)	2 (40%)
** Neurologic Disease**	0 (0%)	1 (17%)	0 (0%)	0 (0%)	1 (20%)
** Pulmonary Disease**	1 (7%)	0 (0%)	1 (12%)	3 (33%)	0 (0%)
** Renal Disease**	0 (0%)	0 (0%)	0 (0%)	2 (22%)	0 (0%)
** Smoker (Current)**	1 (7%)	0 (0%)	1 (12%)	1 (11%)	0 (0%)
** Smoker (Former)**	2 (13%)	2 (33%)	3 (38%)	2 (22%)	1 (20%)
** Former Infection^&^**	2 (13%)	2 (33%)	0 (0%)	3 (33%)	0 (0%)
Treatment (Cancer)					
** Cell Transplant**	3 (20%)	3 (50%)	0 (0%)	0 (0%)	0 (0%)
** Chemotherapy**	12 (80%)	5 (83%)	5 (63%)	7 (78%)	4 (80%)
** Immuno (Anti-CD20)**	1 (7%)	1 (17%)	0 (0%)	0 (0%)	0 (0%)
** Immuno (Anti-CD38)**	1 (7%)	2 (33%)	0 (0%)	0 (0%)	0 (0%)
** Immuno (PD-1 Axis)**	1 (7%)	0 (0%)	0 (0%)	0 (0%)	1 (20%)
** Radiotherapy**	2 (13%)	0 (0%)	2 (25%)	5 (56%)	2 (40%)
** Surgery**	4 (27%)	0 (0%)	5 (63%)	5 (56%)	4 (80%)
** Targeted Therapy**	0 (0%)	0 (0%)	1 (12%)	2 (22%)	0 (0%)
Cancer Status					
** Under Investigation**	5 (33%)	2 (33%)	1 (12%)	0 (0%)	1 (20%)
** Stable Disease**	5 (33%)	1 (17%)	4 (50%)	8 (89%)	3 (60%)
** Remission**	4 (27%)	1 (17%)	0 (0%)	0 (0%)	1 (20%)
** Progression**	1 (7%)	2 (33%)	3 (38%)	1 (11%)	0 (0%)

*Considering alive vs. deceased patients (Mann-Whitney test).

^#^Considering alive vs. deceased patients to verify if death outcome is influenced by age (Fisher’s exact test).

^$^Considering alive vs. deceased patients, comparing individuals with ECOG scores 0-2 vs. 3-4 (Fisher’s exact test).

^&^Refers to pulmonary infections or chronic infections of the urinary system.

Bold values indicate p<0.05.

### Patients with hematologic malignancies that presented leukopenia, lower anti-SARS-CoV-2 antibody production, and higher expression of inflammatory factors went on to die from COVID-19

**Figure 2:**
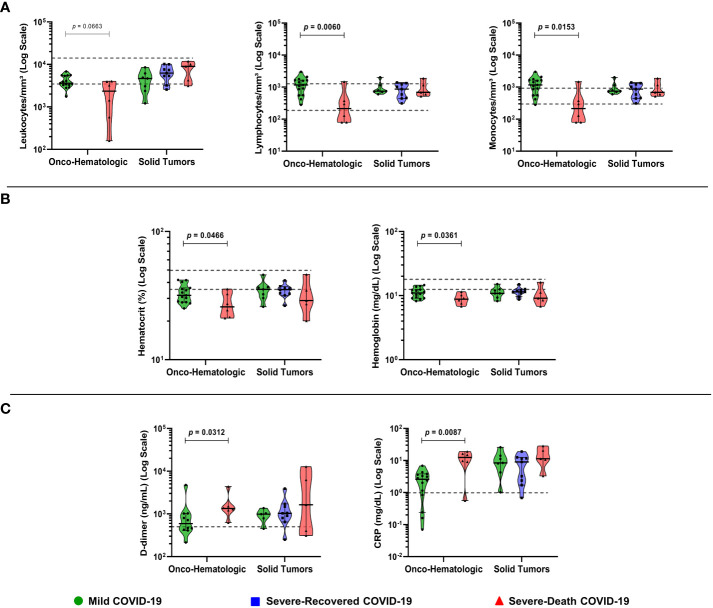
Biochemical and hematological analysis of plasma reveals that low numbers of leukocyte subpopulations are associated with death in patients with hematologic malignancies. Blood samples were collected within two days (median; 1 – 4 CI) of the positive SARS-CoV-2 diagnosis, while hemogram and biochemical exams data were gathered from electronic health records. The graphs depicts leucocytes and subpopulations **(A)**, hematologic parameters **(B)**, and biochemical parameters **(C)**. Groups were defined based on disease severity, as Mild (green), Severe-Recovered (blue), or Severe-Death (red), as described. Gray dashed lines indicate the reference value ranges for each exam. Data is presented as median plus 95% confidence intervals and was analyzed with Kruskal-Wallis adjusted for multiple comparisons followed by Dunn’s *post-hoc* test or Mann-Whitney test. All exact *p*-values below 0.1 are shown. CRP, C-Reactive Protein; N = 21 for Onco-hematologic and N = 22 for Solid tumors group.

To address the potential impact of hematological alterations on COVID-19 progression and outcome, we first evaluated biochemical and hematological parameters assessed at the time of the SARS-CoV-2 infection diagnosis **(** **Figure 2** **)**. Patients with hematologic malignancies that presented with leukopenia, which reflected a decrease in lymphocytes and monocytes **(** **Figure 2A** **)**, went on to die from COVID-19 (Severe-Death group). In addition, hematocrit and hemoglobin values were also lower in Severe-Death hematologic patients, as compared to Mild **(** **Figure 2B** **)**, while neutrophil and platelet numbers did not differ between groups (not shown). Conversely, biochemical analysis showed augmented levels of CRP and D-dimer in the Severe-Death hematologic cancer patients as compared to the Mild group **(** **Figure 2C** **)**, whereas none of the hematological or biochemical parameters differed between solid tumor patients with distinct clinical outcomes **(** **Figures 2A–C** **)**.

**Figure 3:**
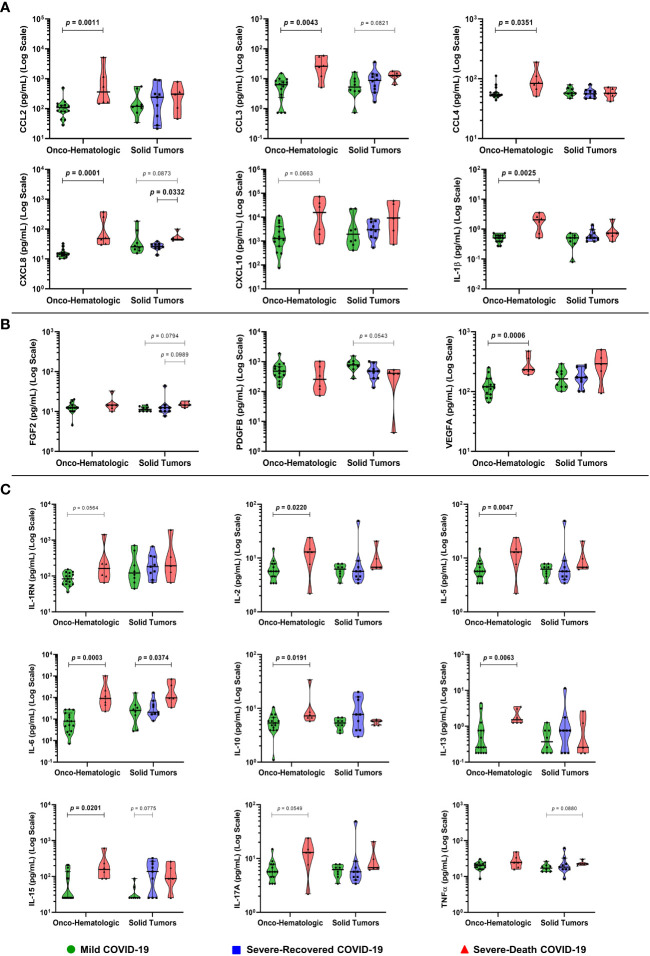
Patients with hematologic malignancies and COVID-19 that display an exacerbated soluble immune profile develop severe disease culminating in death. Blood samples were collected within two days (median; 1 – 4 CI) of the positive SARS-CoV-2 diagnostic and analyzed for 27 immune mediators, being them recruitment factors **(A)**, growth factors **(B)**, and cytokines **(C)**. Groups were defined based on disease severity, as Mild (green), Severe-Recovered (blue), or Severe-Death (red), as described. Data is presented as median plus 95% confidence intervals and was analyzed with Kruskal-Wallis adjusted for multiple comparisons followed by Dunn’s *post-hoc* test or Mann-Whitney test. All exact *p*-values below 0.1 are shown. IL-1RN, Interleukin 1 Receptor Antagonist. N = 21 for Onco-hematologic and N = 22 for Solid Tumors group.

Next, we aimed to better understand the mechanisms related to disease evolution and clinical outcome. To do so we evaluated circulating soluble mediators in plasma sampled within a median of two days of SARS-CoV-2 positive diagnostic **(** **Figure 3** **)**.

Within the hematologic cancer patient cohort there was a striking increase in the levels of the chemokines CCL2, CCL3, CCL4, CXCL8, and the cytokine IL-1β, in the Severe-Death group compared to the Mild group **(** **Figure 3A** **)**. Levels of VEGFA and most of the cytokines measured (IL-2, IL-5, IL-6, IL-10, IL-13, IL-15, IL-17A and IL-1RN) were also increased or showed similar trends in Severe-Death hematologic patients as compared to Mild, with no differences in FGF2, PDGFB, and TNFα concentrations **(** **Figures 3B, C** **)**. No difference was observed for CCL5, CCL11, Colony Stimulating Factor 2 (CSF2), Colony Stimulating Factor 3 (CSF3), IFN-γ, IL-7, IL-9, as well as IL-12A or IL-4, critical for T_H_1 and T_H_2 subset polarization, respectively **(** **Supplementary Figure 2** **)**.

Conversely, patients with solid tumors had fewer changes in the levels of soluble factors than that observed in the hematologic group **(** **Figure 3** **)**, displaying higher concentrations of CXCL8 among patients that went to die compared to those who recovered **(** **Figure 3A** **)**. The concentration of IL-6 was increased in patients with severe disease that progressed to death as compared to Mild, while a similar trend was observed for CCL3, CXCL8, FGF2, and TNFα **(** **Figures 3A–C** **)**. The growth factor PDGFB presented a trend toward a decrease in concentration comparing patients with mild disease and those who would die from COVID-19 **(** **Figure 3B** **)**. In addition, IL-15 showed a trend to increase in patients that had severe disease and recovered, as compared to those in the Mild group **(** **Figure 3C** **)**.

**Figure 4:**
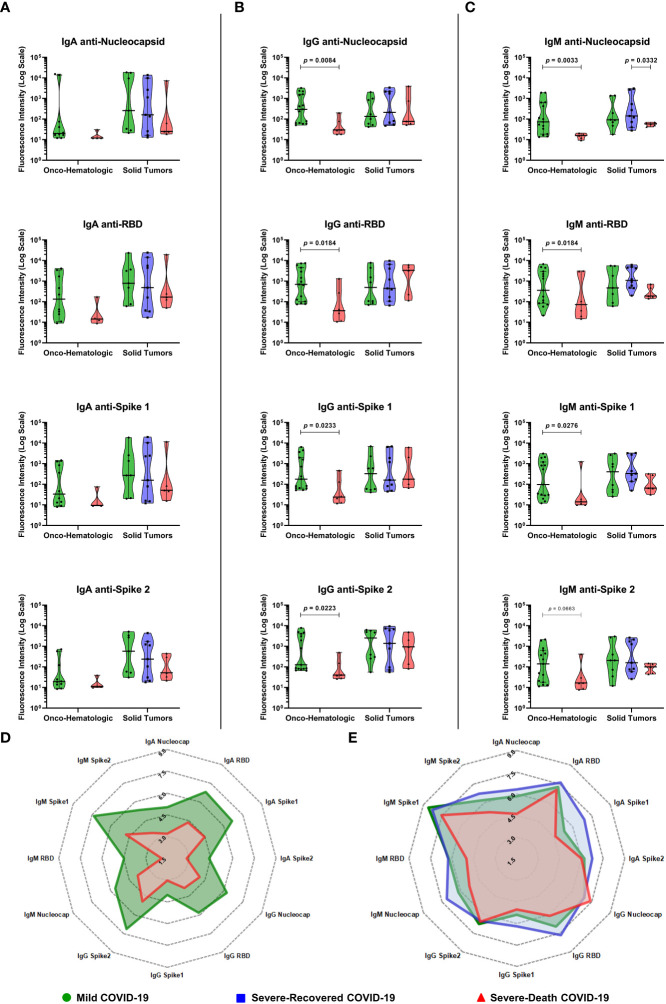
COVID-19 positive patients with hematologic malignancies that develop severe disease culminating in death display decreased IgG anti-SARS-CoV-2 antibodies before disease progression. Blood samples were collected within two days (median; 1 – 4 CI) of the positive SARS-CoV-2 diagnostic and analyzed for Immunoglobulins IgA **(A)**, IgG **(B)** and IgM **(C)** anti-SARS-CoV-2 proteins as described in Materials and Methods. Radar charts for Onco-hematologic **(D)** or Solid Tumors **(E)** patients show the mean values for each factor after fluorescence intensity data was normalized by Ln(x+1). Groups were defined based on disease severity, as Mild (green), Severe-Recovered (blue), or Severe-Death (red), as described. Data is presented as median plus 95% confidence intervals and was analyzed with Kruskal-Wallis adjusted for multiple comparisons followed by Dunn’s *post-hoc* test or Mann-Whitney test. All exact *p*-values below 0.1 are shown. Nucleocapsid, RBD, Spike 1 and Spike 2 labels refers to IgG antibodies anti-SARS-CoV-2 proteins. RBD, Receptor Binding Domain. N = 21 for Onco-hematologic and N = 22 for Solid Tumors group (IgG and IgM measurements) and N = 14 for Onco-hematologic and N = 21 for Solid Tumors group (IgA measurements).

Lastly, we determined the levels of IgA, IgG and IgM antibodies against SARS-CoV-2 nucleocapsid, spike proteins (spike 1 and spike 2), and receptor binding domain (RBD) in patients with different tumors and COVID-19 outcomes **(** **Figure 4** **)**. Interestingly, we observed an overall decrease in levels of IgM and IgG anti-SARS-CoV-2 proteins in the hematologic cancer patients that would die from COVID-19 **(** **Figures 4B, C** **)**, while no changes were observed in IgA levels **(** **Figure 4A** **)**. We also observed a dramatic decrease in IgM anti-nucleocapsid comparing individuals who progressed to death with those who cured among the solid tumor patients **(** **Figure 4C** **)**. An overview of the mean antibody production is represented with radar charts for both hematologic malignancies patients **(** **Figure 4D** **)** and solid tumors patients **(** **Figure 4E** **)**, showing a similar pattern of antibody synthesis from different immunoglobulin classes.

### Unsupervised cluster and network analysis identifies immunological parameters that segregate death from other clinical outcomes in patients with hematologic tumors

**Figure 5:**
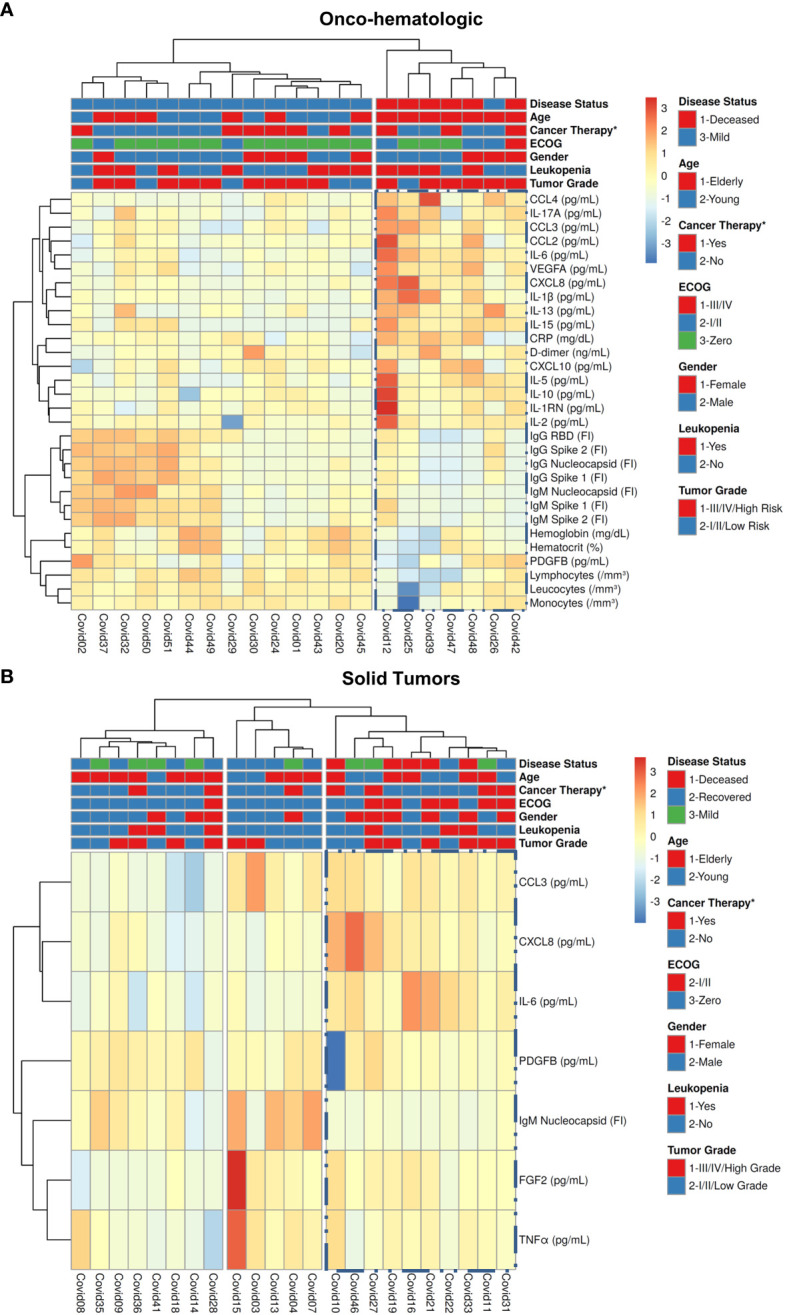
High levels of immune mediators, low IgG anti-SARS-CoV-2, and low leukocyte subpopulations segregate Severe-Death patients with hematologic malignancies from those with Mild disease outcomes. Blood samples were collected within two days (median; 1 – 4 CI) of the positive SARS-CoV-2 diagnostic and analyzed for 27 immune mediators and for immunoglobulins anti-SARS-CoV-2 proteins, as described in Materials and Methods. Groups of hematologic cancer patients **(A)** and solid tumors patients **(B)** were defined based on disease severity as described. All data was normalized by natural logarithm + 1 [ln(x +1)] before running an unsupervised cluster analysis and generation of the heatmap using ClustVis platform as described in Materials and Methods. In **(A)** both rows and columns were clustered by Manhattan distance and Complete linkage. In **(B)** both rows and columns were clustered by Euclidean distance and Ward linkage. Nucleocapsid, RBD, Spike 1 and Spike 2 labels refers to antibodies anti-SARS-CoV-2 proteins. CRP, C-Reactive Protein; RBD, Receptor Binding Domain. *Refers to active cancer therapy one month prior to COVID-19 diagnosis.

To identify combinations of factors that segregate among distinct COVID-19 clinical outcomes we performed an unsupervised cluster analysis within each cancer group, considering only factors with a p-value of 0.1 or below. Hematologic cancer patients segregated based on a major cluster of high production of immune mediators in the Severe-Death group, together with lower levels of IgG and IgM anti-SARS-CoV-2 proteins and lower counts of leukocyte subpopulations **(** **Figure 5A** **).** The solid tumor groups separated the Severe-Death patients in one branch characterized by higher production of soluble immune factors and lower production of IgM. This branch also contained patients with other outcomes, whereas the remainder of patients that had mild COVID-19 or recovered from the disease grouped in secondary branches **(** **Figure 5B** **)**. Categorical patient data, such as chemotherapy one month prior to COVID-19 diagnostic, presence of leukopenia, gender, ECOG score, or tumor grade, did not cluster together with disease outcome. A similar segregation of groups was seen using all the factors analyzed in this project **(** **Supplementary Figure 3** **)**.

To further investigate immunoregulatory mechanisms involved in the response against SARS-CoV-2 and differential clinical outcomes, we generated correlation matrices and functional networks between the soluble immune mediators, anti-SARS-CoV-2 antibodies, leukocyte counts and biochemical indicators.

**Figure 6:**
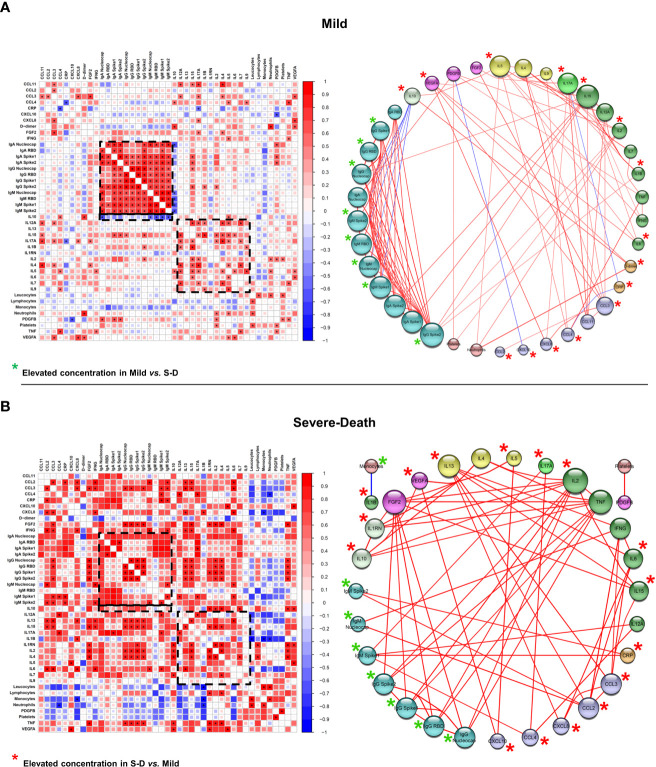
Correlation networks suggest that immune hyperactivation and an impaired antibody response are correlated with death outcome in patients with hematologic malignancies. Data from electronic health records, 27-Plex assay and anti-SARS-CoV-2 proteins’ antibodies assays was used to construct a Spearman’s correlation matrix with all statistically significant (*p* < 0.05, highlighted as *) Spearman’s Rho values, ranging from -1 to 1. The matrix was then used to construct the correlation networks. Red lines connecting the spheres represent positive correlations, whereas line thickness and hotter colors represent correlations with Rho closer to 1. Blue lines connecting the spheres represent negative correlations, whereas line thickness and colder colors represent correlations with Rho closer to -1. Spheres’ sizes correlate with the number of interactions for each factor, whereas the bigger radius represents factors with more interactions. Spheres’ colors represent factors that have similar activities, characteristics or functions. Disease groups were defined as Mild (**A**; n = 15) and Severe-Death (**B**; n = 6), as described. Nucleocapsid, RBD, Spike 1 and Spike 2 labels refers to antibodies anti-SARS-CoV-2 proteins. Monocytes, Neutrophils and Platelets refers to blood counts per mm³ of blood. CRP, C-Reactive Protein; FGF2, Fibroblast Growth Factor 2; IL-1RN, Interleukin 1 Receptor Antagonist; RBD, Receptor Binding Domain.

In patients with hematologic malignancies, we observed striking positive correlations regarding antibody responses in Mild patients, as well as a co-regulation of immunoregulatory cytokines, many of which were missing in those who progressed to death **(** **Figures 6A, B** **)**. Additionally, we identified a negative correlation between the immunoregulatory cytokine IL-10 and general IgM antibody responses in patients with mild disease, which was not observed in those who progressed to death **(** **Figure 6A** **)**. In the Mild group, network analysis also showed several hierarchical interactions, with IL-4, IL-5, IL-12A, IL-15, and IL-17A as major nodes interacting with many immune mediators **(** **Figure 6A** **)**. The cytokine IL-15 showed positive correlations with 13 immune factors, including IL-2, IL-12A, IFN-γ, and anti-SARS-CoV-2 nucleocapsid and spike 2 IgG, classic markers of a T_H_1 type immune response **(** **Figure 6A**; **Supplementary Figure 4A** **)**. The anti-inflammatory cytokine IL-10 increased with increasing levels of IL-5 and D-dimer, indicating a potential coregulation of inflammatory responses. IL-10 also correlated negatively with several IgA and IgM anti-SARS-CoV-2 antibodies. In Severe-Death patients, we observed a bigger impact for interactions formed by the inflammatory cytokines TNF-alpha and IL-2, which had positive correlations with anti-SARS-CoV-2 IgG antibodies, IL-1RN, IL-4, IL-10 and FGF2 **(** **Figure 6B** **)**. Further positive interactions were formed between the inflammatory cytokines IL-6, IL-15 and IFN-γ with different chemokines and IL-13, of which the majority had elevated concentrations in the Severe-Death as compared to the Mild group (indicated by red stars). Lastly, interactions between antibodies decreased among Severe-Death patients in comparison with Mild patients, whereas overall Spearman’s rho values increased with disease severity, highlighting the exacerbated immune response **(** **Figures 6A, B** **;** **Supplementary Figure 4A** **)**. Overall, these findings point to a broad and regulated immune response among hematologic patients who developed mild COVID-19, whereas patients that showed early signs of cytokine-release syndrome, lack of humoral responses and poor immunoregulatory networks progressed to death.

**Figure 7:**
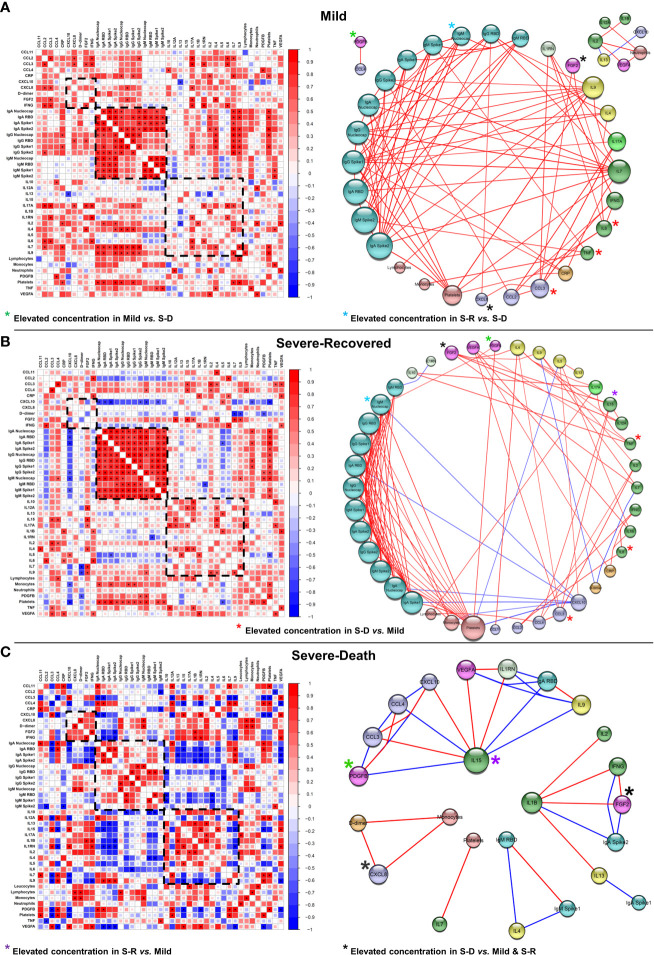
Correlation networks suggest lack of immune regulation and loss of coordinated antibody production in patients with solid tumors that went on to die from COVID-19. Data from electronic health records, 27-Plex assay and anti-SARS-CoV-2 proteins’ antibodies assays was used to construct a Spearman’s correlation matrix with all statistically significant (*p* < 0.05, highlighted as *) Spearman’s Rho values, ranging from -1 to 1. The matrix was then used to construct the correlation networks. Red lines connecting the spheres represent positive correlations, whereas line thickness and hotter colors represent correlations with Rho closer to 1. Blue lines connecting the spheres represent negative correlations, whereas line thickness and colder colors represent correlations with Rho closer to -1. Spheres’ sizes correlate with the number of interactions for each factor, whereas the bigger radius represents factors with more interactions. Spheres’ colors represent factors that have similar activities, characteristics or functions. Disease groups were defined as Mild (**A**; n = 8), Severe-Recovered (**B**; n = 9) and Severe-Death (**C**; n = 5), as described. Nucleocapsid, RBD, Spike 1 and Spike 2 labels refers to antibodies anti-SARS-CoV-2 proteins. Lymphocytes, Monocytes, Neutrophils and Platelets refers to blood counts per mm³ of blood. CRP, C-Reactive Protein; FGF2, Fibroblast Growth Factor 2; IL-1RN, Interleukin 1 Receptor Antagonist; RBD, Receptor Binding Domain.

On the other hand, the networks from solid tumor patients were distinct from those seen in patients with hematologic malignancies, highlighting different immune mechanisms behind COVID-19 progression and outcome **(** **Figures 6** and **7** **)**. Correlation matrices indicate that patients who progressed to mild or recovered from severe COVID-19 presented several positive correlations among the immunological parameters analyzed, which were virtually lost in Severe-Death patients (**Figure 7**). One of those was the striking positive correlations regarding antibody responses in Mild and Severe-Recovered patients, which were not observed in Severe-Death group **(** **Figure 7** **)**. Interestingly, a cluster of positively correlated inflammatory cytokines (IL-1β, IL-2, IFN-γ and FGF2) was present in Severe-Death patients and missing from mild and Severe-Recovered patients **(** **Figure 7** **)**.

Mild solid tumors patients showed a complex network characterized by positive correlations between anti-SARS-CoV-2 antibodies, with IgG and IgA correlating strongly with IL-7, IL-9, IL-4 and platelet counts. In addition, they showed positive networks between anti-inflammatory factors (IL-1RN) and inflammatory factors (IFN-γ, IL-17A, CCL3, and IL-6), suggesting a coregulation of the inflammatory response **(** **Figure 7A** **)**. Moreover, among Severe-Recovered patients, IL-4, IL-12A, IL-15 and IL-17A formed strong positively correlated interactions with one another. IL-4 correlated with other T_H_2 factors, such as CCL11 and IL-9, whereas FGF2 also correlated with T_H_1 factors, such as IL-15, IL-12A, and IL-7 **(** **Figure 7B** **)**. Furthermore, Severe-Recovered patients presented positive correlations specifically between IL-10, IL-17A and TNF, indicating an important regulatory axis. Overall, these findings point to an active complex immunoregulatory network indicative of a balanced type I immune response in solid tumors patients that had mild COVID-19, whereas patients that recovered from severe disease presented an immune profile indicative of mixed type I and type II responses **(** **Figures 7A, B** **)**.

In stark contrast, solid tumor patients that went on to die showed fewer interactions **(** **Figure 7C** and **Supplementary Figure 4B** **)**, characterized by a node composed of CXCL8, D-dimer and monocytes, as well as an IL-15 axis characterized by both strong positive and negative correlations. Most interactions between IgG, IgM or IgA antibodies were lost in comparison with solid tumor patients that progressed to better outcomes. Moreover, almost all interactions between IgA and IgM anti-SARS-CoV-2 proteins and other factors were negative, suggesting that increased immune activity was detrimental to antibody production in those patients **(** **Figure 7** **)**. Lastly, the overall strength of the correlations between factors within the Severe-Death group was stronger than that in the Mild and Severe-Recovered groups **(** **Supplementary Figure 4B** **)**.

### Cytokine levels in early stages of disease act as predictive markers for COVID-19 severity

To investigate the predictive power of soluble immune factors for identifying patients that will go on to severe disease and death (Severe-Death group) *vs*. those that will recover (Mild and Severe-Recovered groups), we performed a random forest analysis, followed by receiver operating characteristic (ROC) curve analysis.

**Figure 8:**
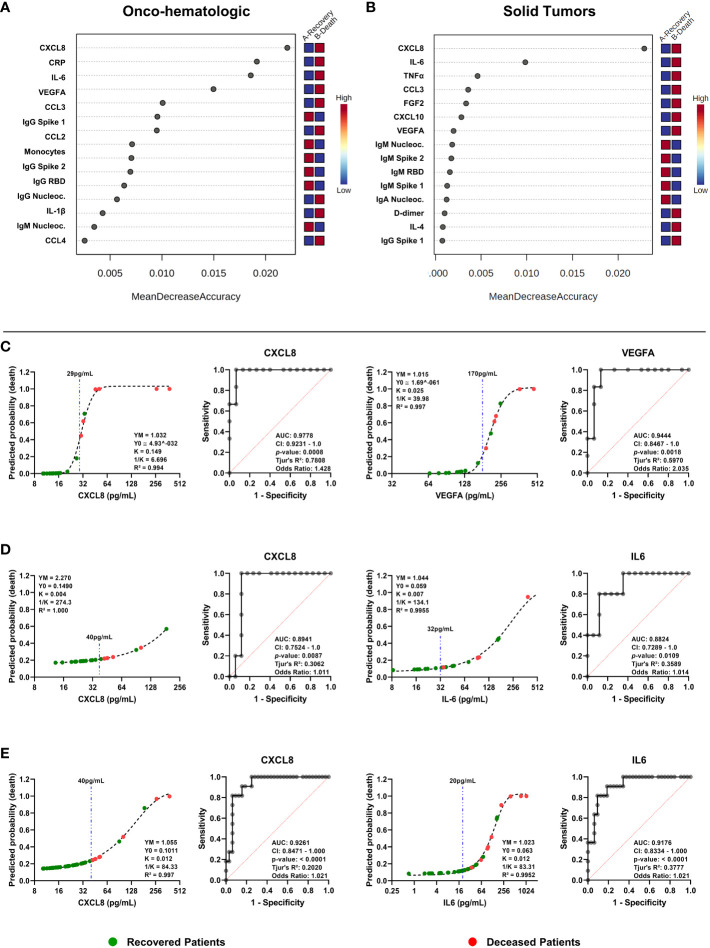
Random forests classification and receiver operating characteristic (ROC) curve analysis indicates CXCL8 as a predictor of death for oncologic patients infected by SARS-CoV-2. Data from electronic health records, 27-Plex assay and anti-SARS-CoV-2 proteins’ antibodies assays was used as a base for random forests classification analysis **(A, B)** or ROC curves construction **(C–E)**. Graphs in **(A)** and **(B)** show the importance of each factor to segregate outcomes of hematologic cancer patients **(A, C)** or solid tumors patients **(B, D)**. ROC curves show area under the curve (AUC), 95% confidence interval of the AUC, *p*-value, Tjur’s pseudo R² and odds ratio for hematologic cancer patients **(C)**, solid tumors patients **(D)**, or both **(E)**. Related line charts show predicted probability of death, R², and variables for the Gompertz Growth model. Dashed blue lines indicate proposed cutoffs in pg/mL. For both groups the outcomes were defined as recovery (indicating all patients that survived) versus death (all patients that went on to die). In **(A, C)**, n = 15 for “Recovery” and n = 6 for “Death”; In **(B, D)**, n = 17 for “Recovery” and n = 5 for “Death”. In **(E)**, n = 22 for “Recovery” and n = 11 for “Death”. Nucleocapsid, RBD, Spike 1 and Spike 2 labels refers to antibodies anti-SARS-CoV-2 proteins. CRP, C-Reactive Protein; RBD, Receptor Binding Domain.

As expected, forest plots showed that patients with hematologic neoplasms had many more segregating factors in comparison with solid tumor patients **(** **Figures 8A, B** **)**. While CXCL8 and IL-6 were the most important factors that segregated Severe-Death solid tumors patients, CXCL8, CRP and IL-6 had important roles for segregation of patients with hematologic cancers **(** **Figures 8A, B** **)**. Interestingly, for both groups antibodies were important factors for segregating patients that had better outcomes, whereas inflammatory factors were important for segregating individuals that went on to die **(** **Figures 8A, B** **)**.

Next, we performed predictive analysis using ROC curves with the top hits identified from the random forest analysis and identified factors that resulted in the highest AUC with clear cutoff values. We also plotted curves in an X by Y graph considering how the immune mediator concentration would influence the probability of death of an individual. Then we applied a Grompetz Growth model, which was the best to explain the data distribution **(** **Figures 8C–E** **)**. We observed that CXCL8 (AUC 0.98) and VEGFA (AUC 0.94) were particularly good at predicting COVID-19 outcomes within hematologic cancer patients **(** **Figure 8C** **)**, while CXCL8 (AUC 0.89) and IL-6 (AUC 0.88) were good predictors for solid tumor patients **(** **Figure 8D** **)**. Finally, we identified both CXCL8 (AUC 0.93) and IL-6 (AUC 0.92) as excellent predictors of death for oncologic patients in general, regardless of whether they had hematologic or solid tumors **(** **Figure 8E** **)**.

## Discussion

**Figure 9:**
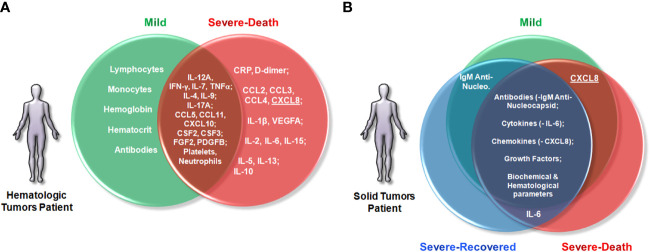
Patients with hematologic malignancies and solid tumors display distinct circulating immune profiles associated with clinical outcomes. Venn diagrams were drawn to summarize the findings of this study, highlighting factors with higher concentrations between the groups. Early increased immune cells and antibody production were associated with mild COVID-19, whereas increased concentrations of inflammatory immune markers were associated with death in the Onco-hematologic group **(A)**. On the other hand, only IL-6 was associated with death among patients with solid tumors. In fact, for those patients, death outcome was associated with a breakdown of coordinate production of antibodies and regulation of soluble factors rather than with broad differences in the concentrations of soluble mediators **(B)**. Lastly, although patients with hematologic and solid tumors display distinct profiles of soluble immune markers, CXCL8 (underlined) was an important early predictive factor of patients that would progress to death from COVID-19 across all cancers. Lymphocytes and monocytes refer to cell counts per mm^3^ of blood. Antibodies refers to IgA, IgG and IgM anti-SARS-CoV-2 Nucleocapsid, Receptor Binding Domain, Spike 1 and Spike proteins. CRP, C-Reactive Protein; IL-1RN, Interleukin 1 Receptor Antagonist.

Our data shows that hematologic and solid tumor cancer patients display distinct systemic immune, hematological and biochemical indicators of disease following infection with SARS-CoV-2, and that these characteristics are capable of segregating patients based on disease outcome **(** **Figure 9** **)**.

First, among blood and biochemical measurements, the hematologic cancer cohort displayed increased CRP and D-dimer concentrations and lower hematocrit and hemoglobin levels in patients that progressed to death (Severe-Death group) **(** **Figures 2B, C** **)**. Although D-dimer evaluation, leukopenia and lymphopenia are useful markers indicative of COVID-19 severity in the general population (15, 16), only the hematologic cancer cohort displayed significant differences in D-dimer concentrations, and lymphocytes and monocyte counts **(** **Figures 2A, C** **)**. However, it is of note that solid tumor COVID-19 patients may also suffer a reduction in specific populations of T cells (17) throughout the disease.

Regarding the systemic soluble immune molecules, the analysis revealed distinct immune networks between hematologic and solid tumor cancer patients, however, both groups displayed elevated concentrations of IL-6 and CXCL8 among individuals that progressed to death **(** **Figure 3** and **5** **)**. The two factors are associated with cytokine-release syndrome and COVID-19 severity (12, 17, 18), and have roles in inflammation, myeloid cell recruitment and tissue damage (19). In our study, they were good early indicators of death risk **(** **Figure 8** **)**.

Among patients with solid tumors, the analysis also showed a trend to increased concentrations of IL-15 in plasma of patients that recovered from the disease compared with patients who only presented mild COVID-19. This increased concentration was significant in patients with hematologic malignancies that would die from COVID-19 compared to individuals that would present only the mild form of the disease **(** **Figure 3C** **)**. Given that NK cells participate in antiviral responses, and that IL-15 is a cytokine with an important role in the proliferation and biology of NK cells (20), it is of interest to determine the role of these cells as inflammatory drivers or infection-resolving agents.

Furthermore, Severe-Death hematologic cancer patients showed a trend to increased production of IL-17A, similar to what is observed in individuals from the general population infected by SARS-CoV-2 and other SARS viruses (21). The Severe-Death hematologic cancer group also had elevated concentrations of IL-5 and IL-13 **(** **Figure 3C** **)**. These factors are associated with eosinophil activation, airway complications, pulmonary diseases, and COVID-19 severity (18, 19, 21), but their exact role in COVID-19 progression or resolution is not well understood.

We also observed increased concentrations of CCL2, CCL3, CCL4, VEGFA and IL-1β in Severe-Death hematologic cancer patients **(** **Figures 3** and **5A** **)**. These factors are important for recruitment of myeloid cells, induction of fever and inflammation (22), and development and restructuring of blood vessels (23). VEGFA specifically is associated with virus spread (24), hypoxia, and tissue-related damage during COVID-19 (25). Overall, patients with hematologic malignancies seem to suffer from exacerbated immune responses, possibly related to the lack of leukocyte subpopulations during the initial phases of infection, since leukopenia is associated with death and the presence of CD8^+^ T Cells with survival (10, 26). Nevertheless, this hypothesis in the oncologic context still needs further investigation.

We also found a striking decrease in the plasma levels of IgG and IgM anti-SARS-CoV-2 proteins in Severe-Death hematologic patients, which was not seen in Severe-Death solid tumor patients **(** **Figure 4** **)**. While antibody titers may vary over the two weeks following the onset of COVID-19 symptoms (27), our findings show that initial antibody levels shortly after diagnosis among hematologic cancer patients indicate possible individuals that would have mild disease, or progress to death **(** **Figures 4** and **5A** **)**, which was also shown in patients from the general population (28).

This lack of effective antibody-based responses among hematologic cancer patients may be associated with myeloma (29), weak T cell responses (30, 31), and oncologic treatments (32). In the COVID-19 context data on this topic is scarce and outcomes are dependent on the type of tumor, treatments, treatment timings, and individual factors (13). The few publications regarding the immune response of patients with cancer against SARS-CoV-2 infections shed light on how chemotherapy, stem cell transplant, and anti-CD20 therapies negatively impact antibody production (33, 34). Nevertheless, we could not observe a clear bias in hematologic malignancy type or cancer treatment in production of antibodies or in the outcome of COVID-19 among our patients **(** **Figure 5**, **Table 1** **)**.

Regarding the multiple correlation analysis, patients with hematologic malignancies that presented only mild disease had extensive networks between a number of immune factors and antibodies, also involving cytokines associated with type I, II and III immune responses **(** **Figures 6A, B** **;** **Supplementary Figure 4A** **)**. We observed prominent roles for type I associated factors, such as IL-2, IL-7, IL-12A, IL-15, and antibodies in general **(** **Figure 6A** **)**, which are known for their importance for combating SARS-CoV-2 infection (25, 35, 36). We also observed a possible regulatory axis for type II responses composed of IL-4, IL-5, and IL-10, and an interesting interaction between IL-17A, CCL4 and CRP **(** **Figure 6A** **)**. Although C-reactive protein is well known for its association with inflammation, it is important to note that this factor also display regulatory proprieties (37).

Regarding Severe-Death hematologic cancer patients, we observed a chemokine axis involving CCL2, CCL3, CXCL8 and IL-6, and an inverse correlation between IL-1β and monocytes **(** **Figure 7C** **)**, indicating that those patients displayed an exacerbated inflammatory response as compared to patients that recovered from COVID-19. We also observed strong correlations involving IL-2, TNF-alpha and immunoglobulins, pointing to an immune response skewed toward myeloid-derived cells, which could further exacerbate the pathological process (25, 38). Supporting this possibility are previous findings indicating that high levels of TNF-alpha and IL-6 correlate negatively with T cell numbers in severe COVID-19 in the general population (30), and that B cell numbers and antibodies are reduced in COVID-19 patients with hematologic malignancies (17). Taken together these results suggest that the lack of a balanced and effective type I lymphocyte-based response, together with an exacerbated inflammatory response, are critical factors leading to death among patients with hematologic malignancies.

On the other hand, network analysis of soluble factors from patients with solid tumors showed that in mild disease IL-1RN forms a potential immunoregulatory network **(** **Figure 7A** **)**. IL-1RN is seen in severe COVID-19 patients from the general population (18), and may have a role counterbalancing inflammatory activity (22) in patients with mild disease. Contrary to what was observed in patients with better outcomes, the Severe-Death group was marked by a negative correlation between IL-4 levels and IgM anti-SARS-CoV-2 proteins **(** **Figure 7C** **)**. In addition, the Mild group displayed a strong correlation between anti-SARS-CoV-2 antibodies, IL-7, and IL-9 that was not seen in the severe disease groups (**Figure 7**). Interestingly, IL-9 shares the γ-chain receptor with other lymphocyte-proliferation cytokines, such as IL-2, IL-4 and IL-7, and aside from its effects on promoting allergic inflammation, IL-9 can also influence the effector activities of T and B lymphocytes, even enhancing the production of IgE and IgG from human cells (39, 40). Conversely, the Severe-Death group was marked by a strong positive correlation between CXCL8, D-dimer and monocytes **(** **Figure 7C** **)**, factors associated with COVID-19 severity among the general population (7, 25, 41, 42), and in solid tumor patients specifically (17). Lastly, we also observed in the Severe-Death group an IL-15-based axis characterized by both strong positive and negative correlations with several immune mediators **(** **Figure 7C** **)**, which highlights the need of a thorough investigation about the role of IL-15 in patients with cancer and COVID-19, given that exhausted T and NK cell populations are observed throughout the disease (17, 28, 35).

Finally, our predictive analysis showed distinctive roles for VEGFA, CXCL8, and IL-6 in segregation of patients by outcome **(** **Figure 8** **)**. None of these factors – with the exception of IL-6 – were important predictors of mortality in studies with large numbers of markers regarding COVID-19 in the general population (18, 43). Therefore, our findings support the need of further studies to validate the use of these factors as potential clinically useful biomarkers.

However, this work has some limitations worth highlighting. The first factor is that cancer itself is a disease with broad manifestations, influenced by several factors, many of which cannot be controlled in a real-life setting. Therefore, we recognize that our cohorts are heterogeneous and that each type of cancer can influence the immune response in particular ways. The second is that cancer therapies also may affect the immune response in particular ways, a situation that depends on several individual factors, one of which is the type of cancer itself. The last is the limited number of patients – a fact present in other studies regarding cancer and COVID-19 (17, 26, 33), and the very specific population (from a Cancer Center in Brazil with acute COVID-19 patients that were all naïve to SARS-CoV-2 vaccines). Importantly, despite these complexities, we identified immune molecules and networks associated with COVID-19 clinical outcome among our cancer patient cohorts.

To the best of our knowledge, this is the first immune-focused study comparing distinct COVID-19 outcomes in patients with cancer. Our results indicate important differences in immune responses in solid tumor and hematologic cancer individuals with COVID-19, suggesting that inflammation based on myeloid-derived cells in early phases of infection could be detrimental for the onset of severe disease and death, as indicated by other studies (25, 41, 42). Given that CXCL8 was an important predictive factor of disease outcome in oncologic patients, it will be interesting to assess if this finding is also observed in other cohorts, and how a therapeutic approach reducing neutrophil-based inflammation could help patients infected by SARS-CoV-2. In addition, investigating specific immune cell populations present in the blood could also help in understanding the different COVID-19 outcomes observed in oncologic patients, especially those with hematologic malignancies.

## Materials and methods

### Patient enrolment and classification

From May to November 2020, we included 43 oncologic patients with a recent diagnostic of SARS-CoV-2 from A.C.Camargo Cancer Center **(** **Table 1** **)**. All volunteer patients received information about the project and gave written consent to participate before initiation of any procedure regarding this work. Individuals were segregated by solid tumors or hematological malignancies based on their previous diagnostic. If the individual had a history of both solid and hematologic tumors, the group selection was based on the most recent or active cancer. Severity of COVID-19 was determined based on the World Health Organization’s proposed recommendations (44), as follows: Mild, no oxygen therapy; Severe-Recovered, for patients who had oxygen therapy, but recovered from the disease; and Severe-Death, for patients who had oxygen therapy and went on to die from COVID-19. For patients that received supplemental O_2_, we considered a minimum of two days of therapy. All patients that received supplemental O_2_ therapy also received dexamethasone treatment as recommended by the RECOVERY Collaborative Group study (45). The project was approved by A.C.Camargo Cancer Center Ethics Committee (CAE:30283220.5.0000.5432).

### SARS-CoV-2 infection assessment

Patients were assessed for infection by SARS-CoV-2 using a real-time qPCR test whenever they presented symptoms for the disease (cough, dyspnea, loss of smell and/or taste, fever) or otherwise indicated by the clinical teams.

### Electronic health records assessment

Biochemical evaluation (C-Reactive Protein and D-dimer concentrations), hematological data (leukocyte, lymphocyte, monocyte, neutrophil, and platelet counts, hemoglobin concentration and hematocrit), tumor classification, Eastern Cooperative Oncology Group (ECOG) performance status scale score, treatment, and other relevant information were obtained from health records available for each patient.

### Blood sampling and processing

Blood samples were collected in EDTA tubes within a median of two days (95% CI: 1 – 4 days) from positive SARS-CoV-2 test result. Blood was centrifuged at 400x*g*, 10 minutes, 20°C for collection of plasma, which was then snap frozen in dry ice and stored at -80°C until further processing.

### Quantification of plasma soluble mediators

Concentration of plasma soluble factors were determined using a 27 multiplex bead assay (Bio-Plex^®^ Pro Human Cytokine Group I Panel 27-plex, Bio-Rad Inc., USA). Multiplex bead assays (Bio-Plex^®^ Pro Human IgA/IgG/IgM SARS-CoV-2 N/RBD/S1/S2 4-Plex Panel, Bio-Rad Inc., USA; provided as three separate kits) were used to measure production of IgA, IgG and IgM antibodies anti-SARS-CoV-2 Nucleocapsid, Receptor Binding Domain (RBD), Spike 1 and Spike 2 proteins. Virus inactivation was performed through incubation at 56°C/30 minutes before analyte quantification. All procedures were done accordingly to the manufacturer’s instructions, and plates were read in Bio-Plex platform (Bio-Plex^®^ 200 system, Bio-Rad Inc., USA). All data is presented as pg/mL, based on standard curves provided by the kit (27-Plex), or as fluorescence intensity (4-Plex antibodies assays).

### Statistical analysis

All soluble factors and hematological data were analyzed in Graph Pad Prism 8.4.2 for construction of Receiver Operator Characteristic (ROC) curves, line plots and scatter dot plots. For scatter dot plots, data from patients with hematologic malignancies was analyzed with a two-tailed Mann-Whitney test, whereas solid tumors patient data was analyzed with a two-tailed Kruskal-Wallis adjusted test followed by Dunn’s *post-hoc*. Data is presented as median and 95% confidence interval.

ROC curves were constructed using the multiple logistic regression model Logit[P(Y=1)] = Ln[(P(Y=1)/P(Y=0)] = β0 + β1*B. Related line charts show data points indicating how analyte concentration influences the probability of death of an individual, calculated based on the model aforementioned. A fitting line was drawn based on the Gompertz Growth Model given in Graph Pad Prism software, with the following equation: Y = YM*(Y0/YM)^(exp(-K*X)), where Y0 is the starting population, YM is the maximum population, K determines the lag time and 1/K is the X value of inflection point.

For radar charts, raw data (fluorescence intensity) from 4-Plex antibodies assays, including values from the positive controls, were transformed by Ln(X+1)*10. Then each measurement of antibody production for each patient was divided by the positive control value of that class of antibody. This way we could correct distinct measurements for different classes of antibodies and all measurements fit inside a scale from 1 to 10. The graphs were constructed in Microsoft Office Excel 2010.

Unsupervised cluster analysis was performed using the online tool ClustVis (46). Raw data from all parameters or parameters with a *p*-value below 0.10 were uploaded in ClustVis platform and transformed with [ln(x +1)] model. The analysis was performed using a hierarchical cluster algorithm where rows and columns were clustered by Manhattan or Euclidean distance and Ward or Complete linkage. Missing data (< 2% of total data) was estimated by imputation as per default.

Correlation matrices were done in R software version 4.0.0 and R Studio 1.4.1106, with *corrplot* and *Hmisc* packages. Briefly, raw data from each patient for each group in both cohorts was organized into a data frame, and a Spearman correlation test was run. All Spearman’s coefficients are shown in the matrices, but a star indicates correlations with a *p-value* of 0.05 or below.

Networks were constructed using the Cytoscape software version 3.8.2. Data from correlation matrices was used to make the networks, considering only interactions with a *p-value* below 0.05. Line thickness and colors refers to strength and value of the interaction, while sphere radius reflects the number of interactions.

For random forests analysis, raw data was uploaded in MetaboAnalyst 5.0 platform (https://www.metaboanalyst.ca) and missing values (< 2% of total data) were estimated by Bayesian Principal Component Analysis. Forest plots were made randomly with 500 trees and 7 predictors to determine the most important factors for segregation accuracy based on outcomes, which were “Recovery” – for all patients that survived; or “Death” – for all patients that died.

## Data availability statement

The raw data supporting the conclusions of this article will be made available by the authors, without undue reservation.

## Ethics statement

The studies involving human participants were reviewed and approved by A.C.Carmaro Cancer Center Ethics Committee (CAE:30283220.5.0000.5432). The patients/participants provided their written informed consent to participate in this study.

## Author contributions

KG and MB conceptualized and designed the study. Data collection was performed by FP-O, AF, NG and KM. Data analysis and interpretation was performed by FP-O, AF, NG, WD, MB and KG. The article was drafted by FP-O, AF, WD and KG. Critical revision of the article was performed by all authors. All authors contributed to the article and approved the submitted version.
