# Supplementary material for: Distinct systemic immune networks define severe vs. mild COVID-19 in hematologic and solid cancer patients
Source: Front Immunol. 2023 Jan 9;13:1052104. doi: 10.3389/fimmu.2022.1052104 (PMC9868546; doi:10.3389/fimmu.2022.1052104)
Supplement: Supplementary file 1 [file DataSheet_1.pdf]

Supplementary Figure 1

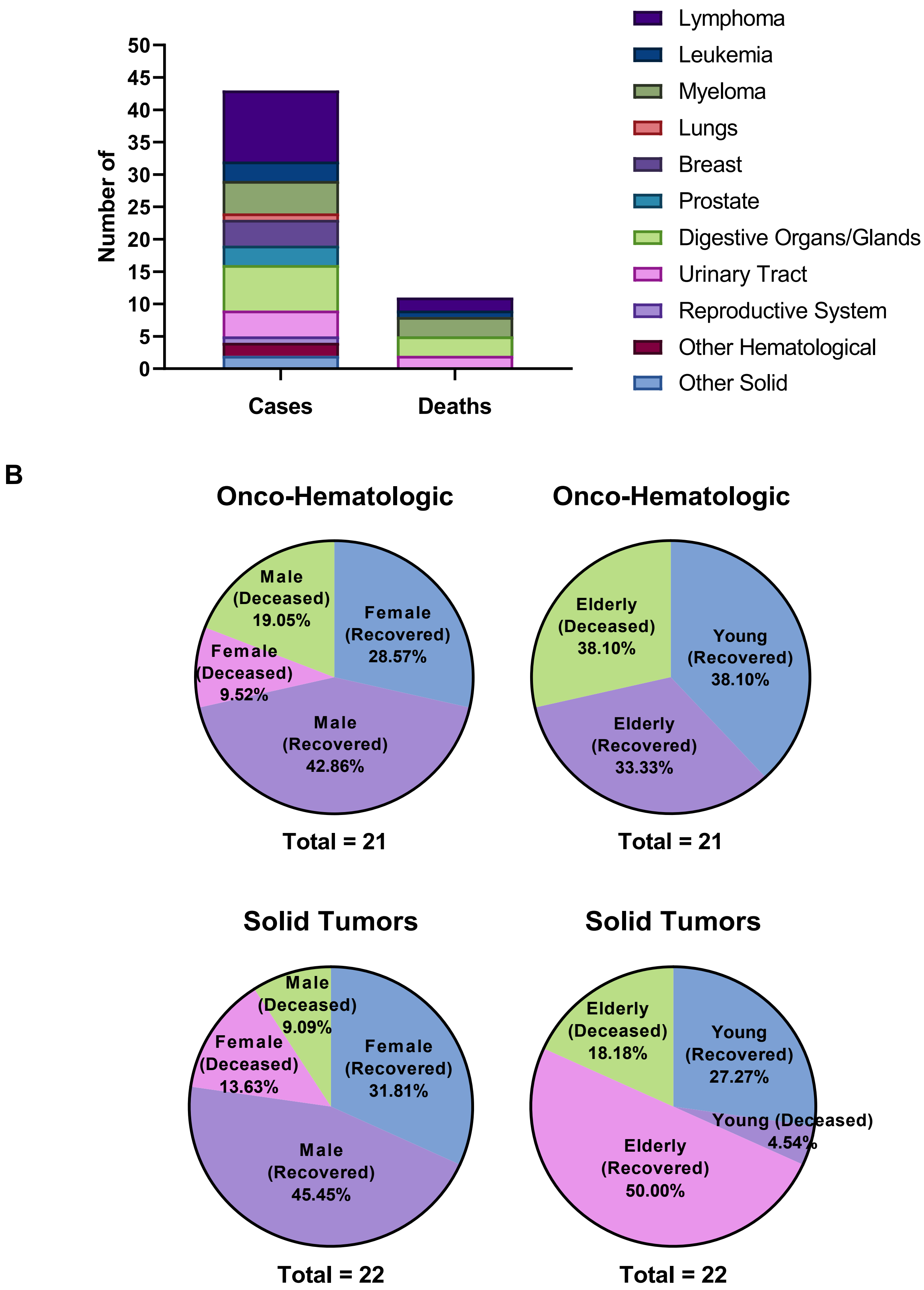

Supplementary Figure 1
Epidemiological data from COVID-19 cancer patients. Graphs show the total number of cases and deaths for each hematological or solid tumour (**A**) or sex and age distribution (**B**) for onco hematological or solid tumours patients. Groups were defined based on disease severity, as Mild, Severe-Recovered, or Severe-Death as described. Data regarding sex and age distribution within each group was analyzed with Fisher's exact test, but no differences were found considering a *p*-value of 0.05 or below.

Supplementary Figure 2

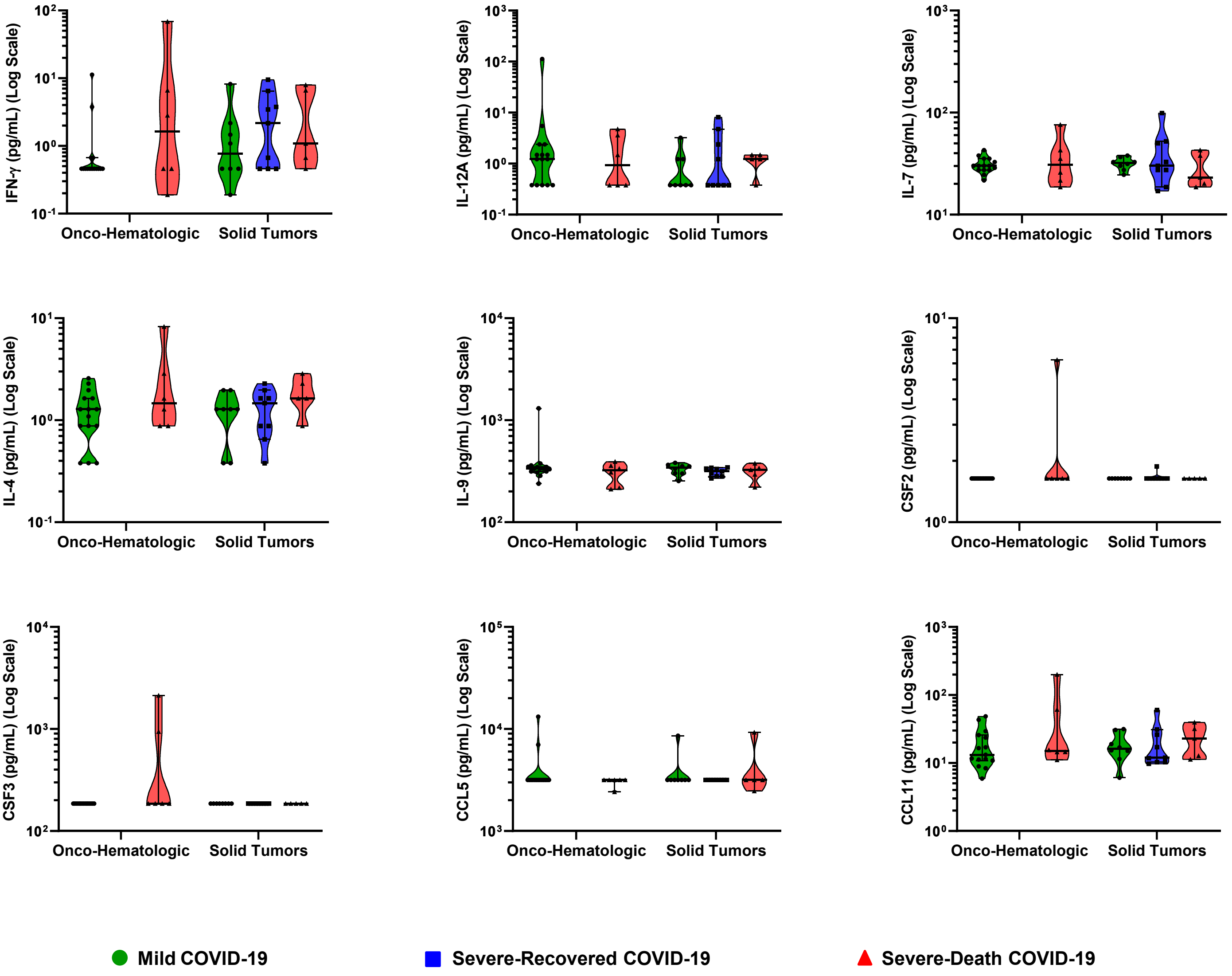

Supplementary Figure 2
Analysis of plasma analites in onco-hematological and solid tumours patients infected by SARS-CoV-2. Blood samples were collected within two days (median; 1 – 4 CI) of the positive SARS-CoV-2 diagnostic and analyzed with a 27-Plex assay or an assay for capturing antibodies anti-SARS-CoV-2 proteins as per manufacturer's recommendations. Groups were defined based on disease severity, as described in Materials and Methods. Colors represent Mild (green), Severe-Recovered (blue), and Severe-Death (red) groups. Data is presented as median plus 95% confidence intervals and was analyzed with Kruskal-Wallis adjusted for multiple comparisons followed by Dunn's post-hoc test or Mann-Whitney test. All exact *p*-values below 0.1 are shown. NS = Not Significant. N = 21 for Onco-hematologic and N = 22 for Solid Tumors.

A

Onco-hematologic

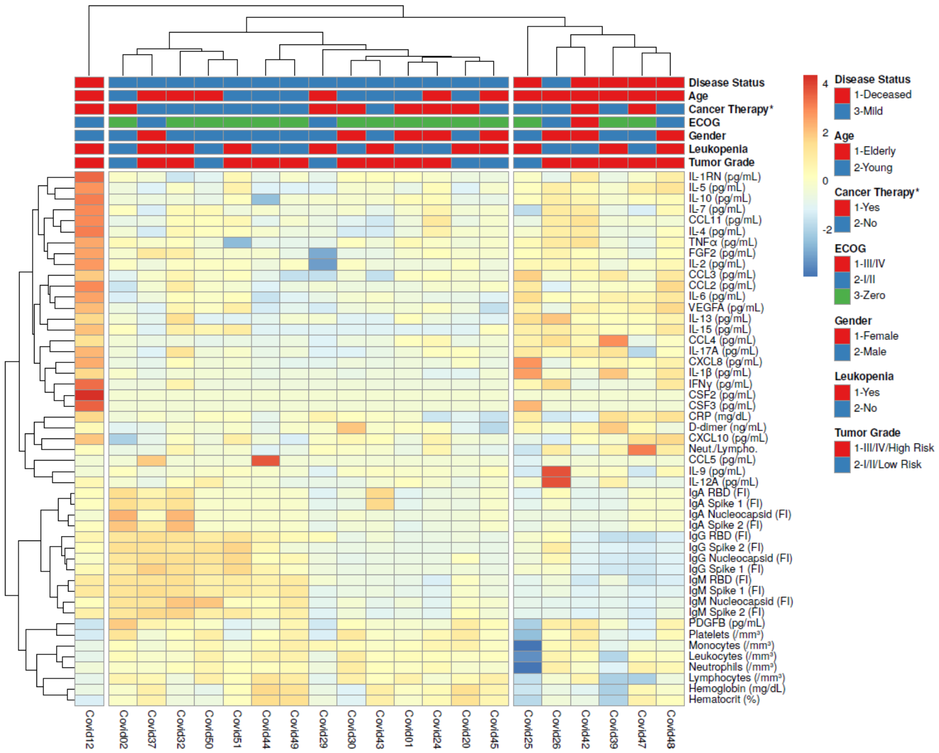

B

Solid Tumors

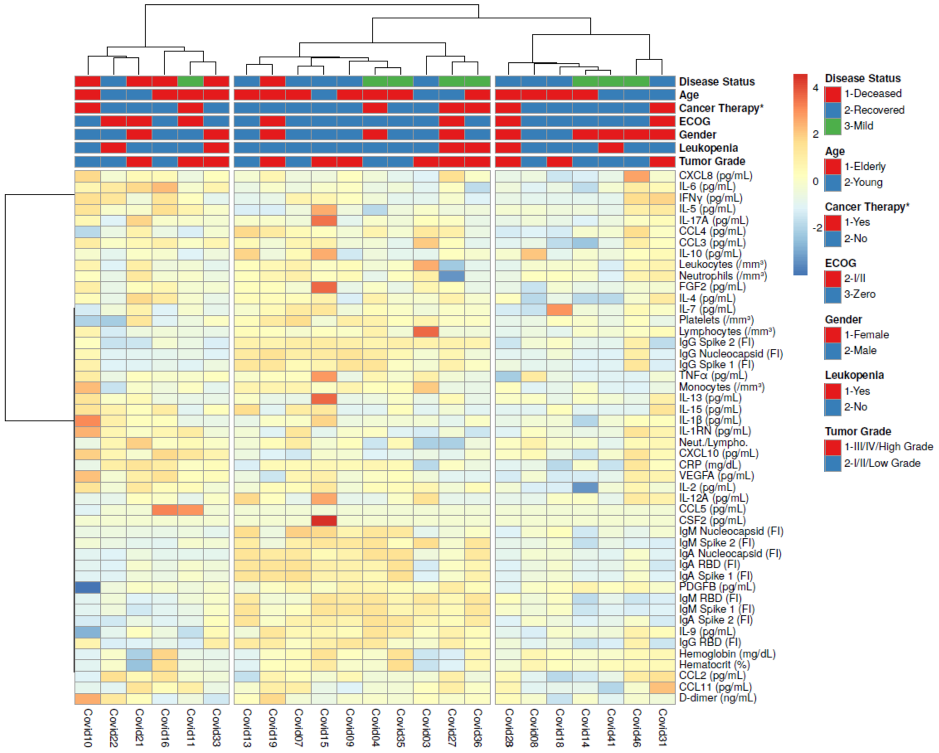

Supplementary Figure 3

Heat map representation of biochemical, hematologic and soluble analites patients in infected by SARS-CoV-2. Blood samples were collected within two days (median; 1 – 4 CI) of the positive SARS-CoV-2 diagnostic and analyzed for 27 immune mediators and for IgG anti-SARS-CoV-2 proteins as described in Materials and Methods. Groups of Onco-hematologic (A) and Solid Tumors patients (B) were defined based on disease severity as described. Clustering was done using all data regarding biochemical, hematological, soluble factors and antibodies. All data was normalized by natural logrythm + 1 [ln(x +1)] before running an unsupervised cluster analysis and generation of the heat map using ClustVis platform as described in Materials and Methods. In (A), both rows and columns were clustered with Manhattan distance and Complete linkage. In (B), both rows and columns were clustered with Canberra distance and Average linkage. Nucleocapsid, RBD, Spike 1 and Spike 2 labels refers to antibodies anti-SARS-CoV-2 proteins. CRP = C Reactive Protein; IL1RN = IL-1 Receptor Antagonist; RBD = Receptor Binding Domain.

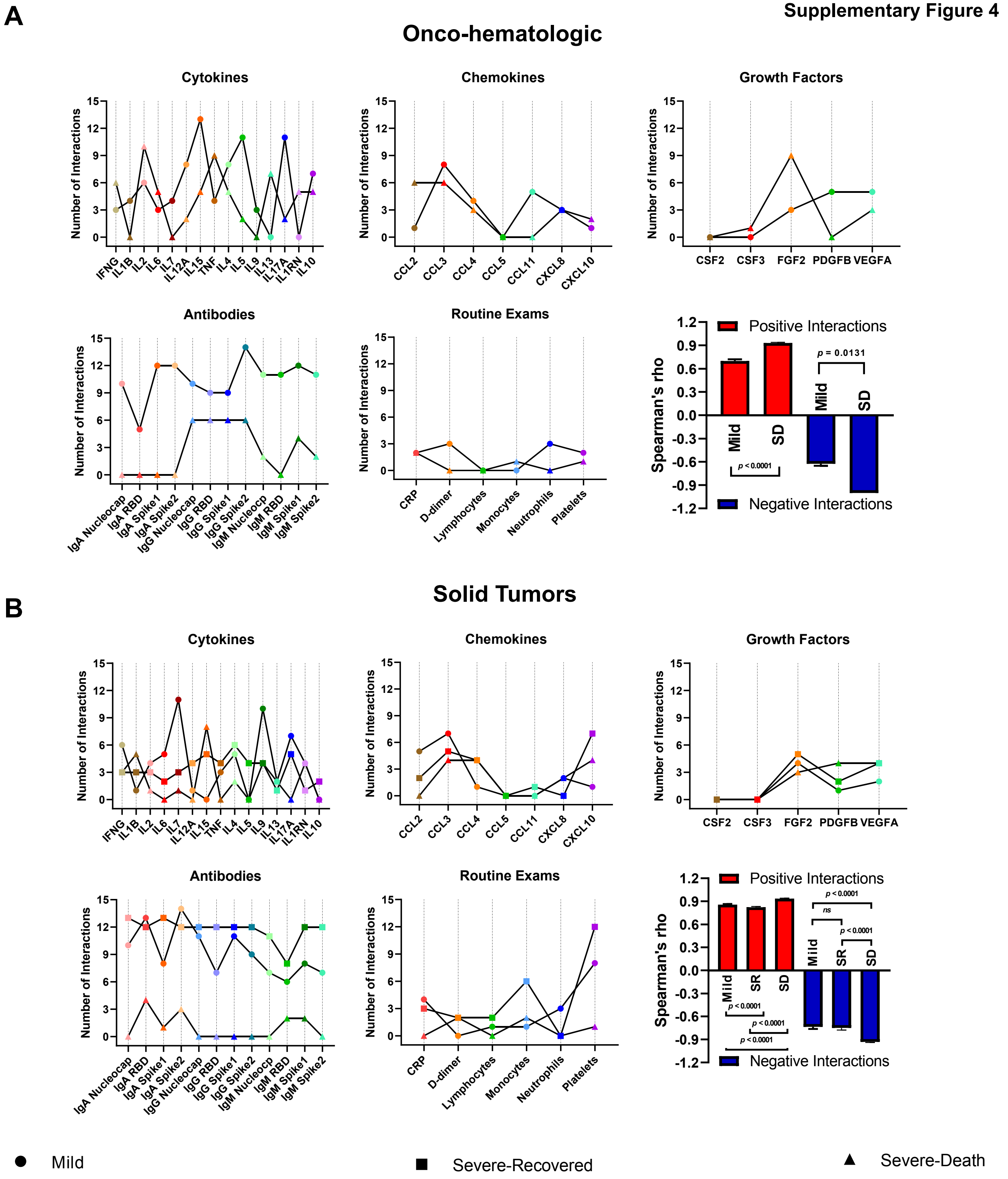

Supplementary Figure 4

Number of correlations decrease as strength of correlation increases for progressively worst outcomes in solid tumours patients infected by SARS-CoV-2. Data from medical records, 27-Plex assay and anti-SARS-CoV-2 proteins' antibodies assay gathered within two days (median; 1 – 4 CI) of SARS-CoV-2 diagnostic was used to construct a Spearman's correlation matrix, considering only interactions with a p-value below 0.05. Graphs represent total number of positive and negative correlations for each group of factors, while the last graph shows the median and 95% confidence interval for Spearman's Rho. Groups were defined based on disease severity, as follows described in Materials and Methods. In (A) are shown the interactions for onco-hematological patients, and in (B) for solid tumours patients. Nucleocapsid, RBD, Spike 1 and Spike 2 labels relate to antibodies anti-SARS-CoV-2 proteins. CRP = C Reactive Protein; FGF2 = Fibroblast Growth Factor 2; IL1RN = Interleukin 1 Receptor Antagonist; PDGFB = Platelet-Derived Growth Factor B; RBD = Receptor Binding Domain.
